# Successful perinatal management of a ruptured brain arteriovenous malformation in a pregnant patient by endovascular embolization followed by elective cesarean section: a single-case experience

**DOI:** 10.1186/s40981-016-0045-6

**Published:** 2016-08-11

**Authors:** Satoru Asano, Nahoko Hayashi, Shunsuke Edakubo, Maiko Hosokawa, Junko Suwa, Yutaka Saito, Shunsuke Ichi, Masuzo Taneda, Keiichi Katoh

**Affiliations:** 1 Department of Intensive Care Medicine, Japanese Red Cross Medical Center, 4-1-22, Hiroo, Shibuya-ku, Tokyo 150-8935 Japan; 2 Department of Anesthesiology, Japanese Red Cross Medical Center, 4-1-22, Hiroo, Shibuya-ku, Tokyo 150-8935 Japan; 3 Department of Neurosurgery, Japanese Red Cross Medical Center, 4-1-22, Hiroo, Shibuya-ku, Tokyo 150-8935 Japan

**Keywords:** Brain arteriovenous malformation, Pregnancy, Cesarean delivery, Endovascular embolization

## Abstract

**Background:**

Although brain arteriovenous malformations (AVM) usually remain asymptomatic during pregnancy, they can cause intracranial hemorrhage and lead to serious neurological deficits. Nowadays, it is accepted that treatment of a ruptured brain AVM during pregnancy should be based on neurologic, not obstetric, indications.

Recently, endovascular treatment has been recognized as a treatment option associated in pregnant patients with brain AVMs.

**Case presentation:**

A 34-year-old woman presented at 25 weeks of gestation with a history of severe headache followed by severe consciousness disturbance. Brain CT showed a subcortical hematoma in the right occipital lobe along with bilateral intraventricular hematomas. A cerebral angiogram was performed to confirm the diagnosis, which revealed right occipital AVM. At 27 weeks of gestation, endovascular embolization of the AVM was attempted under general anesthesia. The feeding artery and the nidus were simultaneously obliterated by injection of 50 % *n*-butyl-cyanoacrylate. As a result, the blood flow into the nidus was drastically decreased and the risk of re-bleeding was substantially reduced. At 38 weeks of gestation, elective cesarean section was performed to deliver the baby under combined spinal-epidural anesthesia (CSEA). An infant weighing 3665 g was delivered, with Apgar scores of 8 and 9 at 1 and 5 min, respectively.

Postoperative analgesia was provided by a continuous infusion of ropivacaine via the epidural catheter. The infant was confirmed as not having any congenital anomalies.

On POD 5, both of the patient and the infant were discharged home without any medical problems. The mother has shown no evidence of re-bleeding from the intracranial lesion since, and the infant is thriving well.

**Conclusions:**

Endovascular treatment in pregnant women is associated with various unique concerns. However, it can be carried out safely and effectively and is useful not only for saving the mother’s life but also for allowing the pregnancy to continue to term.

## Background

Although brain arteriovenous malformations (AVM) usually remain asymptomatic during pregnancy, they can cause intracranial hemorrhage and lead to serious neurological deficits. It is widely accepted that treatment of a ruptured brain AVM during pregnancy should be based on neurologic, not obstetric, indications [1]. However, direct open surgery entails the risk of intraoperative bleeding with deterioration of the uterine and placental circulation and consequent risk also to the fetus [2]. Recently, endovascular treatment, as a non-surgical approach, has been increasingly recognized as a treatment option associated with a lower risk of re-bleeding in pregnant patients with brain AVMs [2, 3]. Recent advances in the devices used in endovascular treatment in conjunction with the development of various embolic materials have encouraged this trend [2, 4].

We report a case of successful perinatal management in a pregnant patient with a ruptured brain AVM by endovascular embolization followed by elective cesarean section. The patient recovered without any neurological deficits, and the infant is thriving well.

## Case presentation

**Fig. 1:**
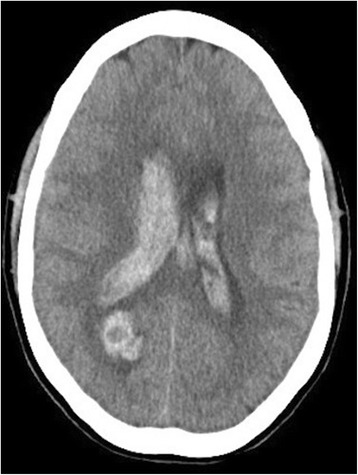
CT image showing a subcortical hematoma in the right occipital lobe, along with bilateral intraventricular hematomas

A 34-year-old multipara woman, with a body weight of 66 kg and measuring 167 cm in height, presented to a neighborhood hospital at 25 weeks of gestation with a history of severe headache of sudden onset followed by severe consciousness disturbance. On the arrival at the hospital, she was assessed as E1V1M5 on the Glasgow Coma Scale (GCS). Her vital signs were normal: blood pressure (BP) 105/59 mmHg, heart rate (HR) 69 bpm, and percutaneus oxygen saturation (SpO_2_) 100 % on room air. Brain computed tomography (CT) showed a subcortical hematoma in the right occipital lobe along with bilateral intraventricular hematomas, which were judged as being a result of rupture of a brain AVM (Fig. 1). Fetal ultrasound revealed a fetus matched for the gestational age, weighing 965 g. Ventricular drainage was urgently performed to control the intracranial pressure and avoid obstructive hydrocephalus.

**Fig. 2:**
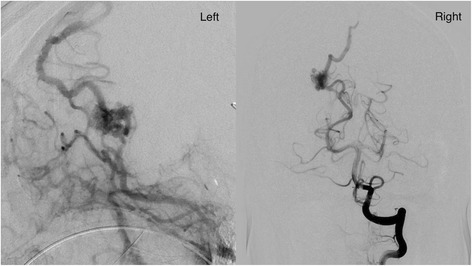
Vertebral angiogram, lateral view (*left*) and anteroposterior view (*right*), showing a grade 2 AVM. The brain AVM measured about 1 cm in diameter (small, 1 point), was located adjacent to the visual cortex (eloquent, 1 point), and drained into the superior sagittal sinus (superficial drainage only, 0 point)

On postoperative day (POD) 1, the patient’s consciousness had fully recovered and she was assessed on the GCS as E4VtM6. A cerebral angiogram was subsequently performed to confirm the diagnosis, which revealed right occipital AVM; the feeding artery was single and arose from the right posterior cerebral artery, feeding a nidus measuring 1 cm in diameter. The draining vein could not be clearly identified but likely drained into the superior sagittal sinus (Spetzler and Martin grade 2; size of the AVM, 1 point/eloquence of the adjacent brain, 1 point/pattern of venous drainage, 0 point) (Fig. 2).

At 27 weeks of gestation, endovascular embolization of the AVM was attempted under general anesthesia. General anesthesia was induced with propofol 120 mg, rocuronium 50 mg, and remifentanil 0.1 μg/kg/min and maintained with end-tidal sevoflurane concentration 1.5 vol.% and remifentanil 0.05 μg/kg/min. The patient was intubated with a 7.0-mm cuffed tube with no increase of BP and HR and ventilated with 40 % oxygen to maintain end-tidal carbon dioxide pressure between 30 and 34 mmHg. The patient’s abdomen was shielded to minimize exposure of the fetus to radiation. The right femoral artery was punctured, and a microcatheter was advanced into a feeder of the AVM by flow control, not directly into the nidus. The feeding artery and the nidus were simultaneously obliterated by injection of 50 % *n*-butyl-cyanoacrylate (NBCA) using live biplane road-mapping systems. Although a tiny residual nidus continued to stain after the embolization, the procedure was completed, because the blood flow into the nidus was drastically decreased and the risk of re-bleeding was substantially reduced.

The total amount of radiation exposure to the patient’s head was determined to be 1446 mGy, with a fluoroscopy time of 20 min 56 s. A total of 128 ml of iohexol, as a non-ionic iodinated contrast agent, was administered intravenously. After the embolization session and emergence of the patient from general anesthesia, a complete neurological examination was performed to assess the changes from the baseline, which confirmed the absence of any neurological deficit. We also confirmed that the fetal HR (FHR) after the procedure was 140 bpm by Doppler monitor. Her intracranial pressure was estimated to be below 15 cm H_2_O by means of the intraventricular drainage tube. On the 28th day from the rupture of the AVM, the patient was discharged home without any neurological problems. The patient went through the rest of her pregnancy until fetal maturity without any obstetric difficulties.

At 38 weeks of gestation, elective cesarean section was performed to deliver the baby. Anesthesia was induced by combined spinal-epidural anesthesia (CSEA). The epidural catheter was atraumatically threaded at the Th12-L1 epidural interspace, and puncture of the dura at the L3-4 interspace was accomplished in the first attempt. Hyperbaric 0.5 % bupivacaine 2.4 ml was injected with fentanyl 15 mcg. An anesthetic level to T4 was obtained after several minutes, and cesarean section was performed uneventfully. An infant weighing 3665 g was delivered, with Apgar scores of 8 and 9 at 1 and 5 min, respectively. Postoperative analgesia was provided by a continuous infusion of 0.2 % ropivacaine at 4 ml/h via the epidural catheter. The infant was confirmed as not having any congenital anomalies. On POD 5, both of the patient and the infant were discharged home without any medical problems.

One month after the delivery, CyberKnife radiosurgery was performed in the patient as stereotactic irradiation for the residual tiny AVM. The mother has shown no evidence of re-bleeding from the intracranial lesion since, and the infant is thriving well.

### Discussion

Management of a ruptured brain AVM during pregnancy requires a multidisciplinary approach with close cooperation among the neurosurgeon, obstetrician, and anesthesiologist for the neurosurgical intervention and the timing and mode of delivery. The risk of re-bleeding in the same pregnancy has been reported to be about 27 %, associated with a 40 % higher mortality as compared to that in age-matched non-gravid women [5, 6]. Therefore, the principle for goal of management of a ruptured brain AVM is to minimize the risk of re-bleeding until delivery. However, it is difficult to make the optimal decisions for the treatment of coma-producing brain AVMs, as there are no definitive guidelines [7, 8].

Endovascular embolization has been used in increasing frequency for the treatment of brain AVMs, not only as radical treatment but also as treatment to decrease the size of the nidus and reduce the blood flow to prevent re-bleeding and facilitate subsequent surgical resection or radiosurgery later [4, 9, 10].

According to the Food and Drug Administration (FDA) recommendations, no drug is fundamentally safe for pregnant women or fetuses. Especially, iodinated contrast agents cross the human placenta and enter the fetus. However, fetal hypothyroidism caused by intravascular administration of non-ionic iodinated contrast agents has not been reported so far [2]. In the present patient, iohexol was used for the embolization, and no abnormalities were observed in the infant. On the other hand, it remains uncertain whether anesthetic agents and adjuvants have an adverse effect on uteroplacental unit and fetus in human [11]. Therefore, we consider that the anesthesia for the AVM embolization should have been managed with the use of FHR monitoring device such as cardiotocography (CTG) in case of non-reassuring fetal status because some obstetric anesthesia texts recommend FHR monitoring for the viable-age fetus during non-obstetric surgery [11, 12].

Radiation exposure of the fetus is also a concern. The International Commission on Radiological Protection (ICRP) recommends that the maximum dose of radiation to the uterus during pregnancy should be under 100 mGy to minimize the teratogenic risks to the fetus [13]. Therefore, Le et al. suggested that delivering the fetus prior to embolization of a brain AVM is preferable to ensure minimal exposure of the fetus to radiation and contrast agents [14]. On the other hand, Tanaka et al. reported that the gonad was exposed to only 0.05–0.07 mGy (0.01 % of the maximum radiation dose to the head) when the head was exposed to a radiation dose of 800 mGy. The radiation dose to the gonad was thus far below than 100 mGy limit recommended by the ICRP, and the author concluded that the risk of radiation exposure of the fetus associated with endovascular treatment of brain AVMs is remarkably low [15]. According to the Spetzler and Martin grading system, the AVM of the present patient was judged as being grade 2 and as being indicated for surgical resection [16]; however, it was deep-seated in the brain and not located in an easily accessible area (Fig. 2). Taking all these data into consideration, it was considered that endovascular embolization rather than resection would be beneficial in the present patient. The radiation dose to the patient’s head was determined to be 1446 mGy, which means that the exposure level of the uterus was 0.14 mGy at the maximum, which was less than the recommended limit by the ICRP.

The anesthetic goal must be to provide strict hemodynamic stability and stable intracranial pressure (ICP) during cesarean section [14, 17]. Hypertension should be avoided to decrease the risk of re-bleeding [8]. Although general anesthesia is described as acceptable in pregnant patients with brain AVMs, hemodynamic instability caused by endotracheal intubation, extubation, and emesis is considered to be disadvantageous. Increased ICP associated with positive airway pressure ventilation is also problematic. On the other hand, spinal anesthesia would provide a sympathetic blockade and allow a pregnant patient to remain awake during cesarean section so that the anesthesiologist can monitor the neurological functions. Although one of the most common complications of spinal anesthesia, especially in parturients, is the serious leakage of cerebrospinal fluid (CSF) from the dura hole which results in decrease of ICP, no previous cases of brain AVM re-bleeding by decrease of ICP induced by CSF drainage have been reported in such patients. Epidural anesthesia is also useful to provide postoperative analgesia in order to avoid cardiovascular responses to pain and reduce the risk of re-bleeding [17, 18].

## Conclusions

Endovascular treatment in pregnant women is associated with various unique concerns, such as the effects of radiation and diagnostic/therapeutic agents on the fetus. However, it can be carried out safely and effectively and is useful not only for saving the mother’s life but also for allowing the pregnancy to continue to term.

## Consent

Written informed consent was obtained from the patient for publication of this case report and any accompanying images. A copy of the written consent is available for review by the Editor-in-chief of this journal.

## Abbreviations

AVM, arteriovenous malformation; BP, blood pressure; CSEA, combined spinal-epidural anesthesia; CSF, cerebrospinal fluid; CT, computed tomography; CTG, cardiotocography; FDA, Food and Drug Administration; FHR, fetal heart rate; GCS, Glasgow Coma Scale; HR, heart rate; ICP, intracranial pressure; ICRP, International Commission on Radiological Protection; NBCA, *n*-butyl-cyanoacrylate; POD, postoperative day; SpO_2_, percutaneus oxygen saturation
